# Neonatal Outcomes Associated with Maternal-Factor-Related Diagnoses: Real-World Evidence from a National Inpatient Database (2010–2025)

**DOI:** 10.3390/medsci14050591

**Published:** 2026-09-19

**Authors:** Tímea Csákvári, Zsuzsanna Kívés, Réka Vajda, Krisztina Palkovics, Ilona Karácsony, Annamária Pakai, Diána Elmer

**Affiliations:** 1 Department of Health Economics and Health Care Management, Institute of Health Insurance, Faculty of Health Sciences, University of Pécs, H-8900 Zalaegerszeg, Hungary; 2 Institute of Health Insurance, Faculty of Health Sciences, University of Pécs, H-7621 Pécs, Hungary; 3 Doctoral School of Health Sciences, Faculty of Health Sciences, University of Pécs, H-7621 Pécs, Hungary; 4 Department of Prevention and Perinatal Medicine, Institute of Basics of Health Sciences, Midwifery and Health Visiting, Faculty of Health Sciences, University of Pécs, H-7621 Pécs, Hungary; 5 Institute of Emergency Care, Pedagogy of Health and Nursing Sciences, Faculty of Health Sciences, University of Pécs, H-7621 Pécs, Hungary

**Keywords:** pregnancy, complications, maternal exposure, neonates, low birth weight, adverse outcomes

## Abstract

Background/Objectives: Maternal conditions and lifestyle exposures strongly influence neonatal morbidity, but population-level longitudinal data from Central and Eastern Europe remain limited. This nationwide study evaluated 16-year trends (2010–2025) in neonatal hospital episodes with maternal-factor-related diagnostic codes (ICD-10 P00, P04, and P70.0–P70.1) in Hungary and quantified associations with severe clinical attributes. Methods: In a retrospective study of 1,546,439 hospital birth episodes, administrative registry data were analysed. Cases with ICD-10 codes P00.0–P00.9 (maternal conditions), P04.0–P04.9 (noxious influences/lifestyle), and P70.0–P70.1 (carbohydrate disorders) were identified. Severity levels, birth weight, surgical interventions, and mechanical ventilation were derived from the Hungarian Diagnosis-Related Group (h-DRG) labels. Temporal trends were evaluated using Joinpoint regression, and episode-level prevalence ratios (PR) with 95% confidence intervals were estimated compared with neonates without recorded maternal factor–related ICD-10 diagnoses. Results: Maternal-factor codes were present in 145,571 episodes (9.41%; P70: 49.96%, P00: 31.33%, P04: 18.71%). Case rates per 1000 live births increased for P04 (7.08-fold), P00 (6.88-fold), and P70 (2.59-fold; overall AAPC: 9.98%, 95% CI: 9.12–10.85). Maternal-factor episodes had a higher prevalence of severe morbidity (PR = 2.18, 95% CI: 2.13–2.23), birth weight 1500–1999 g (PR = 2.27, 95% CI: 2.20–2.34), major surgery (PR = 2.18, 95% CI: 1.86–2.56), and mechanical ventilation > 5 days (PR = 5.04, 95% CI: 4.62–5.51). Mean length of stay increased by 11.05% in P04 episodes (from 4.14 to 4.60 days) and decreased by 15.59% and 23.60% in P00 and P70.0–P70.1 episodes, respectively. Conclusions: Maternal-factor conditions were recorded in nearly one in ten neonatal hospital delivery episodes in Hungary, showing an increase in recorded episode rates and resource use. These coded episodes are associated with a higher prevalence of low birth weight, major surgery, and prolonged mechanical ventilation.

## 1. Introduction

Maternal health—encompassing pre-existing medical conditions, health-related behaviours, and environmental exposures—is a primary determinant of neonatal morbidity and mortality worldwide. Global epidemiological evidence demonstrates that a substantial proportion of adverse perinatal outcomes, including preterm birth, low birth weight (LBW), and stillbirth, are directly attributable to maternal risk factors [1,2,3]. Over recent decades, the epidemiological landscape of maternal health has shifted significantly, driven by an escalating burden of chronic diseases, changing lifestyle patterns, and persistent social inequalities.

Among pre-existing chronic conditions, pregestational and gestational diabetes mellitus have shown alarming upward trends globally [4,5], a pattern mirrored in Central and Eastern Europe [6]. Despite advancements in perinatal care, diabetes-complicated pregnancies remain associated with severe neonatal sequelae, including macrosomia, congenital anomalies, metabolic disturbances, and perinatal death [7]. Concurrently, hypertensive disorders of pregnancy—comprising chronic hypertension, gestational hypertension, pre-eclampsia, and eclampsia—frequently lead to placental insufficiency, resulting in elevated risks of preterm birth, small-for-gestational-age birth, NICU admission, and long-term neurodevelopmental or cardiometabolic deficits in offspring. Crucially, when maternal hypertension and diabetes co-occur, they exhibit additive or synergistic adverse effects, substantially increasing the risk of severe complications such as neonatal seizures compared to either condition alone [8].

Behavioural determinants, particularly maternal substance use, further compound neonatal risk. Tobacco smoking during pregnancy remains a leading preventable cause of LBW and preterm birth [9,10]. Prenatal alcohol exposure likewise poses critical threats; heavy drinking causes fetal alcohol spectrum disorders, while no safe threshold for low-to-moderate consumption has been established. Furthermore, the rapid escalation of illicit drug use—especially opioids—has driven a dramatic rise in neonatal abstinence syndrome (NAS). In the United States, NAS hospitalizations nearly doubled between 2010 and 2017 [11]. NAS is strongly associated with prolonged NICU stays, respiratory complications, and neurobehavioral impairment in childhood, often occurring within a broader context of polysubstance use and socioeconomic deprivation [9,12,13,14,15].

Environmental exposures and broader social determinants of health are superimposed on clinical and behavioural factors. Ambient air pollution, indoor smoke from solid fuels, and occupational hazards contribute substantially to LBW and adverse fetal outcomes [16]. These hazards disproportionately affect socioeconomically disadvantaged and racially marginalized populations, who may also experience a higher burden of maternal chronic conditions and health-related risk factors. Consequently, persistent social and structural inequalities contribute to disparities in neonatal outcomes, highlighting the complex interplay between biological, behavioural, environmental, and structural determinants [2,17].

Despite the growing international evidence on maternal risk factors and neonatal outcomes, population-based longitudinal analyses integrating maternal diseases, lifestyle-related exposures, and neonatal severity indicators remain scarce in Central and Eastern Europe. Furthermore, no nationwide Hungarian study has simultaneously examined temporal trends, disease severity, healthcare resource utilization, and adverse neonatal outcomes within a single analytical framework. Findings from North America or Western Europe cannot be directly extrapolated to Hungary, where health system structures, baseline risk profiles, and social determinants differ. Between 2010 and 2025, Hungary underwent marked demographic changes characterised by population decline, low but fluctuating fertility and population ageing [18]. However, robust longitudinal evidence evaluating whether temporal trends in neonates ill due to maternal causes diverge from the total newborn population remains scarce. Moreover, few studies in the region have evaluated pre-existing diseases and maternal lifestyle factors within a single, unified epidemiological framework.

The present study therefore aims to answer three interrelated research questions for Hungary between 2010 and 2025. First, we investigate how the annual number and recorded episode rate of neonatal hospital episodes coded with maternal-factor-related diagnoses (ICD-10 P00, P04, P70) have changed over time, and whether these trends differ from the total hospital-born newborn population. Second, we compare the occurrence of severe neonatal outcomes (e.g., low birth weight, major surgery, prolonged ventilation) between neonate episodes with maternal-factor-attributed codes and those without. Third, we evaluate whether specific diagnostic subgroups (P00: maternal conditions; P04: noxious influences/lifestyle; P70: carbohydrate disorders) differ in their associated clinical severity and resource use.

## 2. Materials and Methods

### 2.1. Study Design and Setting

This study was a nationwide, registry-based, population-level, retrospective secondary data analysis covering the period 2010–2025.

### 2.2. Data Sources and Study Population

Nationwide administrative data were obtained from the Pulvita Healthcare Data Warehouse of Hungary. This integrated system compiles a unique, large-scale dataset based on records from the National Health Insurance Fund Administration, which is the sole public health insurer in Hungary. In addition, it incorporates financing information and mandatory in-area care data from the National Center for Public Health and Pharmacy for specialist services, supporting health policy planning and decision-making. Access to and use of these anonymised data are regulated and authorised by the National Directorate General for Hospitals.

A second data source was the Hungarian Central Statistical Office (KSH), from which we obtained national live birth counts for each calendar year between 2010 and 2025. Using these data, we calculated the number of cases per 1000 live births for the overall neonatal population and for each diagnostic subgroup separately.

### 2.3. Case Definition and Diagnostic Classification

For the maternal factor–related neonatal subgroup, we identified all cases with ICD 10 codes P00.0–P00.9 (“Fetus and newborn affected by maternal conditions that may be unrelated to present pregnancy”; e.g., maternal hypertensive disorders, renal and urinary tract diseases, infectious and parasitic diseases, circulatory and respiratory diseases, nutritional disorders, injury, surgical procedures and other medical procedures on the mother) (P00), P04.0–P04.9 (“Fetus and newborn affected by noxious influences transmitted via placenta or breast milk”; e.g., maternal anaesthesia and analgesia or other medication, maternal use of tobacco, alcohol or drugs of addiction, maternal use of nutritional or environmental chemical substances, and unspecified maternal noxious influences) (P04), and P70.0–P70.1 (Syndrome of an infant of a mother with gestational diabetes or pre-existing diabetes) (P70). From the initial 146,036 identified maternal-factor records (P00: 45,813; P04: 27,371; P70: 72,852), 465 episodes (0.32%) grouped by the administrative system into non-neonatal pediatric or surgical DRGs were excluded because standard neonatal clinical attributes (e.g., birth weight categories, neonatal complication tiers) could not be derived from their DRG labels. This yielded a final analytical cohort of 145,571 maternal-factor neonatal episodes for the severity and episode-levelprevalence ratio (PR) analyses. The reference group comprised all remaining neonatal DRG episodes with a type-3 diagnosis of P96.4 but without P00.0–P00.9, P04.0–P04.9 or P70.0–P70.1 in secondary diagnosis fields. This group is therefore defined by the absence of the selected codes, not by the absence of all maternal, obstetric or placental conditions (e.g., ICD-10 P01–P03). The analysis was restricted to P00, P04 and P70.0–P70.1 because these rubrics explicitly attribute the neonatal episode to a maternal medical condition, a noxious influence transmitted via placenta or breast milk, or maternal diabetes. Other perinatal codes were outside the prespecified research questions and were not used to define exposure or reference status.

For each identified episode, we retrieved the calendar year of admission and the full h-DRG label. In addition, we obtained the number of cases, the h-DRG weight, and the number of inpatient days, which allowed calculation of the average length of stay (LOS) as total inpatient days divided by the number of cases within each category. The overall neonatal reference population population comprised all neonatal hospital care episodes recorded in the national inpatient financing database between 2010 and 2025, identified by the presence of code P96.4 as a type-3 diagnosis (diagnosis type ‘3’ denotes the ‘care-justifying main diagnosis‘). This operationalization follows rule of Ministerial Decree 10/2012 (II. 28.) NEFMI, under which the first hospital care of a newborn—irrespective of its actual duration—is classified as newborn hospital care, and the type-3 diagnosis of such an episode can only be P96.4, signaling the first care episode associated with the child’s birth. This P96.4 based population served as the nationwide neonatal baseline against which the distribution of DRG based severity categories in the maternal factor–related subgroup was compared.

### 2.4. Neonatal Outcomes

We derived additional clinical variables from the DRG labels rather than from individual patient records. Specifically, birth weight categories, surgical status (yes/no/only minor abdominal), and the presence and severity of neonatal complications (e.g., none, moderate, severe, other) were inferred from the wording of the h-DRG descriptions; episodes without explicit information on a given attribute were treated as missing for that variable. Mechanical ventilation was identified via h-DRG codes specific to ventilatory support and its duration, and episodes lacking such codes were considered non ventilated, reflecting the strong financial and coding incentives associated with this high-cost intervention in the h-DRG system.

Because the Hungarian DRG system does not provide a standardized neonatal severity classification applicable across all neonatal DRG categories, we developed a predefined severity framework based on the clinical content of the h-DRG descriptions. The classification was established before the statistical analyses and applied consistently to all neonatal episodes throughout the study period. For the classification of composite clinical severity, we grouped h-DRGs into four ordinal categories: 0 (no or minimal problem), 1 (mild problem/low care intensity), 2 (moderate problem/increased care intensity), and 3 (severe problem/high care intensity). Our categorisation was primarily based on the overall clinical content of each DRG label, while the average DRG weight per case was used as supporting information rather than as a financing driven criterion.

DRGs such as very low birth weight (<1500 g), presence of major surgery, need for mechanical ventilation were classified as severe (category 3).

DRGs describing significant but non-critical conditions (e.g., no major surgery, but moderate-level problem/symptom) were classified as moderate (category 2). Also, for the DRGs labelled “newborn transferred before day 5, born in the same hospital” and “newborn transferred before day 5, born elsewhere”, we classified these cases as category 2 (moderate problem/increased care intensity). This decision reflects that such DRGs do not describe routine newborn care, but rather an early need for escalation of care and organised transfer to another unit or institution. In clinical practice, transfer within the first five days of life typically indicates that the infant cannot remain on a normal postnatal ward and requires additional monitoring, diagnostic work up or access to higher level neonatal services (e.g., regional or national centres), which implies increased resource use and clinical concern compared with healthy term newborns. At the same time, these DRGs do not themselves specify a clearly defined, life threatening condition (such as extreme low birth weight, major surgery or prolonged mechanical ventilation), which are captured by other neonatal DRGs that were classified as severe (category 3). To avoid conflating organisational transfer with documented severe morbidity, transfer related DRGs were therefore consistently assigned to the moderate severity category.

DRGs reflecting minor or self-limited conditions (e.g., birthweight between 2000–2499 g, but no major surgery or problem) were classified as mild (category 1).

DRGs indicating normal newborn care (e.g., term newborn with normal diagnosis) were considered to represent no or minimal problem (category 0).

Purely technical codes (e.g., invalid main diagnosis, missing data) and DRGs clearly referring to maternal delivery modes (e.g., vaginal delivery) were recoded as “X” and excluded from analyses of severity categories, because they do not reflect neonatal morbidity or care intensity.

Non neonatal DRGs (e.g., renal, haematological, ENT codes) attached to neonatal ICD 10 P episodes occurred extremely rarely (465 episodes, 0.32% of all DRGs) and were treated as potential coding artefacts or non-primary neonatal conditions; these DRGs were likewise labelled as “X” and excluded from the analytic dataset.

To ensure transparency and reproducibility, the complete allocation of every neonatal h-DRG to its corresponding severity category is provided in Supplementary Table S1.

### 2.5. Statistical Analysis

We first performed descriptive statistical analyses to characterise the nationwide neonatal DRG dataset and the maternal factor–related subgroup. For each calendar year and composite clinical severity level, we calculated absolute case counts, proportions, rates per 1000 livebirths, average LOS (total inpatient days divided by the number of cases).

For the prevalence ratio (PR) analyses, we defined mutually exclusive groups of hospital birth episodes within the national h-DRG dataset. The maternal-factor group comprised all neonatal DRG episodes with a type-3 diagnosis of P96.4 and at least one secondary ICD-10 diagnosis from P00.0–P00.9, P04.0–P04.9 or P70.0–P70.1. The reference group (episodes without the selected maternal-factor codes) included all remaining neonatal DRG episodes with a type-3 diagnosis of P96.4 but without any of these codes in secondary diagnosis fields. The unit of analysis was the hospital episode; estimates are therefore episode-level prevalence ratios (ratios of outcome proportions), not person-level risks in unique neonates. Subgroup PRs for P00, P04 and P70.0–P70.1 were calculated analogously, using all episodes carrying the respective code. These subgroups are not mutually exclusive unique infants. If more than one of these codes was recorded, each code generated a separate episode record; subgroup totals are therefore episode counts.

For each outcome category (birth weight, surgical status, h-DRG-based problem severity and mechanical ventilation), we calculated the outcome proportion in each group as the number of episodes with the given attribute divided by the number of episodes in that group with a valid value for the attribute. PRs were obtained as the ratio of these two proportions, with 95% confidence intervals derived on the log scale using standard binomial approximations. Because the same neonate may contribute more than one episode (e.g., inter-hospital transfer), the binomial intervals assume independence of episodes and may be anti-conservative.

To explore temporal trends, we applied Joinpoint regression to annual indicators derived from the DRG data. Joinpoint models were used to identify significant changes in trend over the 2010–2025 period and to estimate the average annual percent change (AAPC) and segment-specific annual percent changes (APCs) for each time series, based on the breakpoints determined by the software. For each segment, the software provided *p* values for the APC estimates, indicating whether the corresponding trend could be characterised as a statistically significant increase or decrease in the annual percentage change. All statistical tests were two sided, and *p* values below 0.05 were considered statistically significant.

Data management, summary statistics and contingency tables were generated using Microsoft Excel 365 (Microsoft Corporation, Redmond, WA, USA) and R 4.6.1 (R Foundation for Statistical Computing, Vienna, Austria), while Joinpoint regression analyses were performed with Joinpoint Regression Program version 4.9.0.0 (National Cancer Institute, Bethesda, MD, USA) [19].

### 2.6. Ethical Considerations

Both Pulvita and the KSH provided only aggregated datasets that contained no direct or indirect personal identifiers and could not be linked to individual patients. As the study relied exclusively on non-identifiable secondary data and did not involve the use of human clinical records at the individual level, formal approval from a medical research ethics committee was not required.

## 3. Results

### 3.1. Descriptive Statistics

Between 2010 and 2025, a total of 1,546,439 hospital birth episodes were identified by the type-3 ICD code P96.4, of which 145,571 eligible episodes involved neonatal conditions recorded with maternal-factor-related codes, lifestyle factors or other maternal influences (ICD codes P00.0–P00.9, P04.0–P04.9, P70.0–P70.1). The remaining 1,400,868 episodes constituted the reference group.

Among the maternal-factor records, the largest proportion was accounted for by P70 cases (72,720; 49.96%), followed by P00 (45,614; 31.33%) and P04 (27,237; 18.71%). In contrast, when examining the total DRG weight assigned to these subgroups, the highest financed weight remained associated with P70 (49,629.24; 43.79%), and the P00 subgroup ranked second (39,915.06; 35.22%), ahead of P04 (23,800.52; 21.00%).

**Figure 1:**
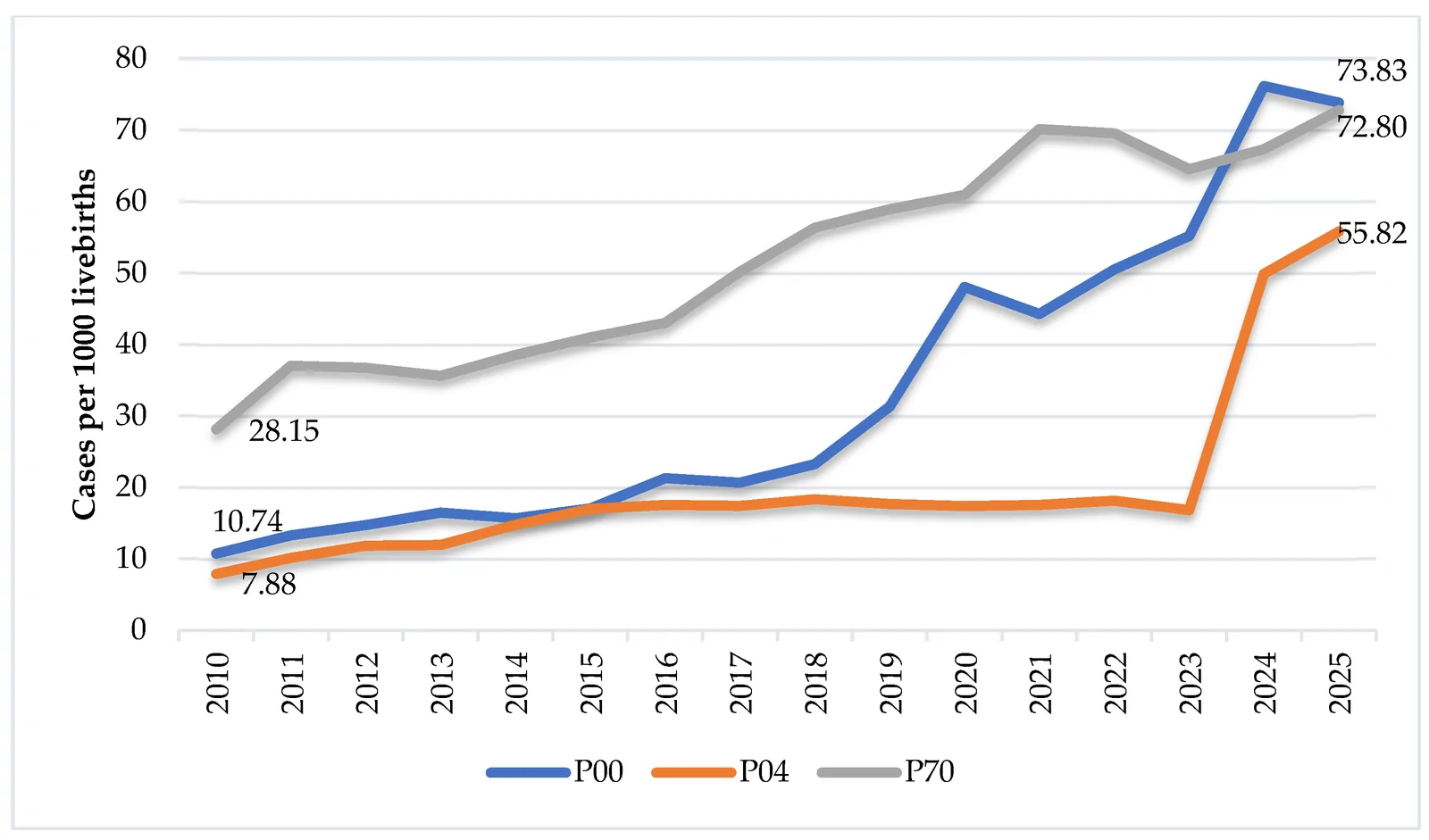
Case numbers per 1000 livebirths for disease groups P00, P04 and P70.

A similar pattern was observed when examining case counts per 1000 live births: trends in these indicators are shown in detail in Figure 1. Overall, the rate per 1000 live births increased for all three diagnostic groups over the study period: between 2010 and 2025, the P04 rate rose 7.08-fold, the P00 rate 6.88-fold and the P70 rate 2.59-fold.

Between 2010 and 2025, the greatest relative increase in total DRG weight was observed for P04, where the cumulative weight rose 7.68-fold (from 530.42 to 4072.56). In the P00 subgroup, the total DRG weight increased 6.36-fold (from 873.16 to 5549.71), while in the P70 subgroup it increased 2.54-fold (from 1641.29 to 4169.31).

Regarding the average LOS, a decrease was observed for the P00 (−15.59%; from a mean of 4.73 to 3.99 days) and P70.0–P70.1 groups (−23.60%; from 4.03 to 3.08 days). By contrast, mean LOS increased modestly in the P04 group (+11.05%; from 4.14 to 4.60 days).

### 3.2. Distribution of Case Severity

We examined which DRGs were coded in association with each ICD category to assess the presence of complications in the recorded episodes. In most cases, one of the neonatal DRGs was assigned, and only a negligible proportion of records contained missing or invalid DRG information or non-neonatal DRGs; these were excluded from this part of the analysis in accordance with the criteria described in the Section 2.

**Figure 2:**
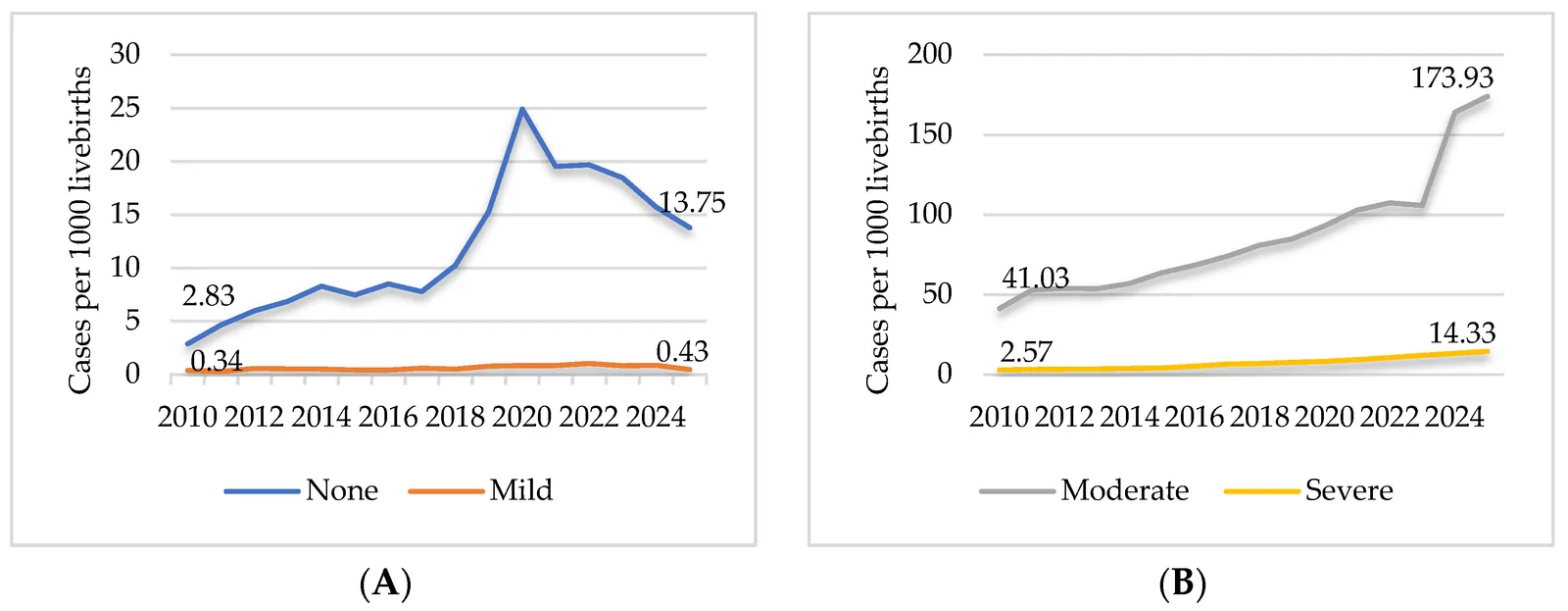
Trends in neonatal hospital episodes per 1000 live births across composite clinical severity categories (2010–2025). (**A**) Low-severity and complication-free episodes (None and Mild); (**B**) Moderate-to-high severity episodes (Moderate and Severe). Note the difference in y-axis scaling between panels to account for high-volume moderate morbidity cases.

Overall, rates per 1000 live births increased in every composite severity category between 2010 and 2025. The largest relative increase was observed for severe episodes (from 2.57 to 14.33 per 1000 live births; 5.58-fold), followed by complication-free (None) episodes (from 2.83 to 13.75; 4.85-fold) and moderate episodes (from 41.03 to 173.93; 4.24-fold). Mild episodes remained rare and increased only modestly (from 0.34 to 0.43; 1.25-fold). Concurrently, absolute episode counts rose substantially for moderate (from 3706 to 12,526; +238.0%) and severe categories (from 232 to 1032; +344.8%), as well as for complication-free episodes (from 256 to 990; +286.7%), whereas mild episodes remained unchanged (31 to 31; 0.0%) (Table 1). The distribution of episode counts per 1000 live births by severity category is presented in Figure 2 (Panel A: None and Mild; Panel B: Moderate and Severe).

Among the four severity categories, mean length of stay declined in all groups. The largest relative reductions were observed in mild (−31.11%; from 4.35 to 3.00 days) and complication-free episodes (−30.83%; from 3.99 to 2.76 days). Mean LOS decreased by 19.56% in the severe group (from 6.63 to 5.33 days) and by 6.86% in the moderate group (from 4.08 to 3.80 days). Detailed severity-specific data are presented in Table 1. In addition, an exhaustive breakdown for each individual ICD-10 subcode is detailed in Table S2.

Joinpoint analyses demonstrated significant overall increases in annual case numbers, rates per 1000 live births, and DRG weights for total neonatal DRG episodes and for most diagnostic and severity groups between 2010 and 2025 (Table S3). For total episodes, the AAPCs were 8.52% for case numbers (95% CI: 7.67% to 9.37%), 9.98% for rates per 1000 live births (95% CI: 9.12% to 10.85%), and 9.41% for DRG weight (95% CI: 8.42% to 10.41%). Total case rates increased by 8.72% annually between 2010 and 2023 and by 18.52% annually between 2023 and 2025. Overall length of stay decreased for total episodes (AAPC: −0.59%; 95% CI: −0.90% to −0.28%), P00 episodes, and P70 episodes, whereas P04 length of stay increased slightly. Mild-group case numbers and rates per 1000 live births showed no significant overall changes, although their segment-specific trends varied over time. Full APC and AAPC estimates are provided in Table S3.

### 3.3. Prevalence Ratio Analysis

The distribution of neonatal clinical outcomes, birth weights, surgical interventions, problem severities, and ventilation requirements among the maternal-factor subgroup compared to the reference group is summarized in Table 2.

Overall, neonatal hospital episodes with maternal-factor-related codes were associated with a higher prevalence of adverse clinical attributes than episodes without these codes. Compared with the reference group, the prevalence of severe composite DRG-defined conditions was more than twofold higher in the maternal-factor group (PR = 2.18, 95% CI: 2.13–2.23; 6.53% vs. 3.00%), and the prevalence of moderate severity was also elevated (PR = 1.60, 95% CI: 1.60–1.61; 81.48% vs. 50.81%). Conversely, an uncomplicated (None) course was substantially less frequent (PR = 0.25, 95% CI: 0.25–0.26; 11.43% vs. 44.92%).

Low-birth-weight categories showed consistently higher prevalence in maternal-factor episodes, with the strongest associations in the 1000–1499 g and 1500–1999 g strata (both PR = 2.27; 95% CI: 2.16–2.38 and 2.20–2.34, respectively). Extremely low birth weight (<999 g) was also more frequent (PR = 1.71, 95% CI: 1.61–1.81). Maternal-factor episodes were further associated with a higher prevalence of major surgery (PR = 2.18, 95% CI: 1.86–2.56) and of mechanical ventilation, particularly ventilation lasting more than 5 days (PR = 5.04, 95% CI: 4.62–5.51). These estimates are unadjusted, episode-level prevalence ratios and should be interpreted as associations between co-recorded diagnostic codes, not as causal effects of maternal exposure.

Disaggregated analyses revealed distinct clinical profiles across the three diagnostic groups. Episodes coded with P00 showed the highest prevalence ratios for severe composite conditions (PR = 3.03, 95% CI: 2.94–3.13), very low birth weight 1000–1499 g (PR = 3.74, 95% CI: 3.51–3.98), extremely low birth weight < 999 g (PR = 3.17, 95% CI: 2.94–3.41), major surgery (PR = 3.02, 95% CI: 2.41–3.80), and prolonged ventilation > 5 days (PR = 5.82, 95% CI: 5.12–6.62).

P04 episodes were associated with a higher prevalence of low birth weight, particularly 1500–1999 g (PR = 3.13, 95% CI: 2.96–3.31) and 2000–2499 g (PR = 2.29, 95% CI: 2.22–2.37), minor abdominal surgery (PR = 4.83, 95% CI: 2.22–10.47), and prolonged ventilation (PR = 3.81, 95% CI: 3.13–4.63).

The P70.0–P70.1 group—accounting for the largest share of maternal-factor episodes—was characterised mainly by moderate composite severity (PR = 1.87, 95% CI: 1.87–1.87) and a higher prevalence of short- and long-duration ventilation (PR = 3.25, 95% CI: 3.04–3.46 and PR = 5.02, 95% CI: 4.49–5.62, respectively). Associations with low birth weight were weaker than in P00 and P04, and extremely low birth weight was less frequent than in the reference group (PR = 0.70, 95% CI: 0.62–0.79). The prevalence of minor abdominal surgery did not differ significantly from the reference group (PR = 1.28, 95% CI: 0.52–3.16).

## 4. Discussion

The primary aim of this study was to characterize the recorded burden and clinical profile of neonatal hospital episodes with maternal-factor-related diagnostic codes (ICD-10 P00, P04, and P70.0–P70.1) in Hungary between 2010 and 2025. These codes were present in 145,571 of 1,546,439 hospital birth episodes (9.41%), corresponding to nearly one in ten recorded episodes. Rates per 1000 live births increased over the study period in all three diagnostic groups, most markedly for P04 (7.08-fold) and P00 (6.88-fold), and more moderately for P70.0–P70.1 (2.59-fold). Compared with episodes without these codes, maternal-factor episodes were associated with a higher prevalence of low birth weight, major surgery, prolonged mechanical ventilation, and moderate or severe DRG-defined conditions. Because exposure and outcome attributes were captured within the same administrative episode and could not be adjusted for individual-level confounders, these findings describe associations between coded hospital episodes rather than causal effects of maternal disease or lifestyle.

Maternal medical conditions such as hypertensive disorders [20], renal disease [21,22], infections [3,23,24,25], and cardiovascular conditions [8,26] are known to increase the likelihood of placental dysfunction, intrauterine growth restriction, preterm delivery, and fetal hypoxia. This literature is compatible with the P00 profile in our data: P00 episodes showed the highest prevalence ratios for very low and extremely low birth weight, major surgery, severe composite DRG category, and ventilation lasting more than 5 days. These associations are biologically plausible, but they may also be amplified by coding practice, because P00 is, by definition, assigned when a newborn is recorded as affected by a maternal condition and is therefore more likely to appear among already complicated episodes.

Noxious influences transmitted via the placenta or breast milk—notably maternal use of tobacco [10,27,28,29], alcohol, or drugs of addiction [30,31]—have long been associated with impaired fetal growth and placental insufficiency. In our cohort, P04 episodes combined a steep rise in recorded rates and DRG weight with elevated prevalence of low birth weight, minor abdominal surgery, and prolonged ventilatory support. Mean length of stay for P04 increased modestly at the group level in Table 1 (from 4.14 to 4.60 days; +11.05%), whereas it declined for P00 and P70.0–P70.1. This pattern is consistent with a greater recorded care intensity among P04 episodes, but it cannot distinguish a true increase in clinical complexity from changes in case mix or coding.

Gestational and pre-existing diabetes classically predispose to macrosomia, neonatal hypoglycaemia, preterm birth, and neonatal intensive care admission [4,7,32]. After restricting the carbohydrate-related exposure to P70.0 and P70.1, this group still accounted for about half of maternal-factor episodes and was dominated by moderate rather than severe composite categories. The comparatively weaker, and for <999 g even inverse, association with extremely LBW is consistent with the known tendency of maternal diabetes toward higher rather than extremely LBW, while the elevated prevalence of mechanical ventilation may reflect metabolic transition, respiratory adaptation, or co-recorded morbidity.

The observed temporal increases in all three diagnostic groups are likely driven by a combination of factors. Rising prevalence of chronic maternal conditions such as hypertension, diabetes, and obesity, together with postponed childbearing and persistent harmful lifestyle patterns, may have increased the underlying burden of maternal morbidity. At the same time, improvements in diagnostic awareness and coding practices, including more consistent recording of maternal-factor ICD codes and better capture of ventilation, surgical, and severity-weighted DRGs, could have contributed to the upward trends in recorded episodes and DRG weights. Administrative data cannot clearly separate changes in true epidemiological incidence from changes in recorded episode rate. Accordingly, the consistent pattern of higher prevalence of low birth weight, moderate and severe complications, major surgery, and prolonged ventilation should be read as evidence of a growing recorded clinical and resource burden, not as proof that disease incidence increased to the same extent. This still supports the practical importance of preconception and antenatal care, control of chronic maternal diseases, and lifestyle interventions, while leaving causal attribution open.

In this context, early identification and prenatal risk stratification of pregnancies at high risk for adverse neonatal outcomes remain a critical challenge. Although recent studies have explored the prognostic utility of first-trimester biochemical (e.g., PAPP-A, free β-hCG) and ultrasonographic markers for predicting neonatal complications such as low Apgar scores or NICU admissions, their standalone predictive accuracy remains modest [33]. Nationwide real-world inpatient data therefore remain useful for describing the downstream recorded clinical and resource burden of maternal-factor-related codes and for informing perinatal healthcare planning.

### Strengths and Limitations

To our knowledge, this is the first investigation in Central and Eastern Europe to examine, in a single framework, the recorded prevalence, temporal trends, and associated outcome profiles of neonatal hospital episodes coded with maternal-factor-related diagnoses. Leveraging a nationwide administrative database over a 16-year period (2010–2025) yielded a large sample of more than 1.5 million hospital birth episodes. Beyond describing temporal trends, the study combines epidemiological analyses with DRG-based measures of recorded severity, healthcare resource utilization, and unadjusted prevalence ratios of adverse neonatal attributes. By providing longitudinal real-world evidence from a Central and Eastern European healthcare system, the findings address an important geographical evidence gap and may support maternal health policies, risk stratification, and the planning of preventive and perinatal healthcare services.

Nevertheless, several limitations should be considered. First, the lack of individual-level deterministic linkage between maternal and neonatal records precluded direct within-cohort comparisons of exposed infants against unexposed controls, as well as adjustment for critical maternal and perinatal confounders (e.g., maternal age, parity, body mass index, smoking intensity, gestational age, multiple pregnancy, or socioeconomic status). Consequently, the reported prevalence ratios represent aggregated, episode-level comparisons, which may be influenced by administrative coding practices and unmeasured confounding. The reference group may still include episodes affected by other obstetric or placental conditions [34]; prevalence ratios therefore contrast selected maternal-factor codes with all other birth episodes, not with a fully unexposed neonatal population. Second, the primary analytical unit was the hospital care episode rather than a deterministically unique neonate. Although the cohort was anchored to birth-related admissions identified by the type-3 diagnosis P96.4, transferred newborns or rehospitalizations could not be tracked across facilities without personal identifiers. If more than one maternal-factor code (P00, P04, or P70.0–P70.1) was recorded, each code generated a separate episode record. Subgroup totals and subgroup PRs are therefore episode counts, not mutually exclusive unique infants. Third, because maternal-factor diagnoses (ICD-10 secondary codes) and neonatal outcome attributes were captured within the same inpatient hospitalization records, the observed associations may partly reflect administrative documentation clustering alongside true biological etiology. The substantial secular increases observed across all three diagnostic categories—particularly the sharp rise in P00 and P04 rates—undoubtedly reflect an interplay between epidemiological change (such as increasing maternal comorbidities and advanced maternal age) and enhanced diagnostic awareness coupled with evolving hospital coding practices. While nationwide administrative data lack external chart-audited re-abstractions to formally quantify coding drift, several factors suggest that documentation artefacts alone cannot fully account for the observed patterns. Specifically, surgical procedures and mechanical ventilation were identified from DRG labels tied to high-cost interventions rather than from diagnostic coding alone. Furthermore, the distinct clinical profiles across P00, P04, and P70.0–P70.1, together with the granular 4-character subcode distributions (Supplementary Table S2), support the internal consistency of the findings, while still precluding causal inference.

## 5. Conclusions

By integrating national routine inpatient data and applying standard epidemiological indicators, this study provides a comprehensive, quantitatively grounded description of neonatal hospital episodes coded with maternal-factor-related diagnoses (ICD-10 P00, P04, and P70.0–P70.1) in Hungary over a 16-year period. These codes were recorded in nearly one in ten hospital birth episodes and became more frequent over time, particularly P00 and P04. Episodes assigned these codes were associated with a higher prevalence of low birth weight, moderate or severe DRG-defined conditions, major surgery, and prolonged mechanical ventilation than episodes without such codes. Disaggregated analyses showed distinct recorded profiles across maternal medical conditions, noxious/lifestyle-related codes, and maternal-diabetes-related carbohydrate codes. Because the data are observational, administrative, and episode-level, the study cannot establish causality and cannot demonstrate that temporal increases in diagnostic coding correspond directly to increases in underlying underlying occurrence of disease. The findings nevertheless provide context-specific real-world evidence from Central Europe that may inform targeted preconception policies, perinatal healthcare planning, and clinical resource allocation.

## Figures and Tables

**Table 1:** Changes of main outcomes regarding composite clinical severity categories from 2010 to 2025.

Variables	Disease Groups	None			Mild			Moderate			Severe			Total		
		2010	2025	Change (%)	2010	2025	Change (%)	2010	2025	Change (%)	2010	2025	Change (%)	2010	2025	Change (%)
Case number (*n*)	P00	185	629	240.00%	20	18	−10.00%	685	4211	514.74%	80	458	472.50%	970	5316	448.04%
	P04	70	361	415.71%	11	13	18.18%	597	3369	464.32%	34	277	714.71%	712	4020	464.61%
	P70	1	-	-	-	-	-	2424	4946	104.04%	118	297	151.69%	2543	5243	106.17%
	Total	256	990	286.72%	31	31	0.00%	3706	12,526	237.99%	232	1032	344.83%	4225	14,579	245.07%
Case number (per 1000 livebirths)	P00	2.048	8.734	326.48%	0.221	0.250	12.89%	7.583	58.472	671.11%	0.886	6.360	618.12%	10.738	73.816	587.44%
	P04	0.775	5.013	546.89%	0.122	0.181	48.24%	6.609	46.781	607.86%	0.376	3.846	921.93%	7.882	55.820	608.22%
	P70	0.011	-	-	-	-	-	26.833	68.678	155.94%	1.306	4.124	215.72%	28.151	72.802	158.62%
	Total	2.834	13.747	385.08%	0.343	0.430	25.44%	41.025	173.931	323.96%	2.568	14.330	457.97%	46.770	202.438	332.83%
DRG weight (amount)	P00	55.74	260.19	366.79%	7.76	11.41	47.04%	306.16	2746.18	796.98%	503.5	2531.3	402.74%	873.16	5549.71	535.52%
	P04	21.62	147.27	581.17%	3.48	9.02	159.20%	297.6	2408.13	709.18%	207.72	1508.14	626.04%	530.42	4072.56	667.80%
	P70	0.32	-	-	-	-	-	1156.35	3139.69	171.52%	484.62	1029.62	112.46%	1641.29	4169.31	154.03%
	Total	77.68	407.46	424.54%	11.24	20.43	81.76%	1760.11	8294	371.22%	1195.84	5069.06	323.89%	3044.87	13,790.95	352.92%
Mean LOS (days)	P00	4.16	2.77	−33.54%	5.40	3.06	−43.42%	4.60	4.05	−11.97%	6.94	5.15	−25.76%	4.73	3.99	−15.59%
	P04	3.54	2.75	−22.52%	2.45	2.92	19.09%	4.08	4.62	13.44%	7.12	6.83	−3.99%	4.14	4.60	11.05%
	P70	3.00	-	-	-	-	-	3.93	3.01	−23.21%	6.28	4.21	−32.92%	4.03	3.08	−23.60%
	Total	3.99	2.76	−30.83%	4.35	3.00	−31.11%	4.08	3.80	−6.86%	6.63	5.33	−19.56%	4.21	3.83	−9.02%

**Table 2:** Prevalence ratio based on certain neonatal outcome variables between the reference group and maternal-factor neonate episodes registered between 2010–2025. (cases where outcome could not be derived from the DRG description were excluded for the PR analysis).

Outcome Variable	Level	Reference n (%)	Maternal-Related n (%) ^a^	Maternal-Related PR (95% CI)	P00 PR (95% CI)	P04 PR (95% CI)	P70.0–P70.1 PR (95% CI)
Severity category ^c^	ref N = 1,400,868 ^b^						
	None	629,333 (44.92)	16,635 (11.43)	0.25 (0.25–0.26)	0.56 (0.55–0.57)	0.42 (0.41–0.43)	0.00 (0.00–0.00)
	Mild	17,803 (1.27)	812 (0.56)	0.44 (0.41–0.47)	0.82 (0.75–0.90)	0.96 (0.87–1.07)	0.00 (0.00–0.01) †
	Moderate	711,725 (50.81)	118,613 (81.48)	1.60 (1.60–1.61)	1.27 (1.26–1.28)	1.45 (1.44–1.46)	1.87 (1.87–1.87)
	Severe	42,007 (3.00)	9511 (6.53)	2.18 (2.13–2.23)	3.03 (2.94–3.13)	2.14 (2.04–2.24)	1.66 (1.61–1.72)
Birth weight	ref N = 1,396,605 ^b^						
	<999 g	7175 (0.51)	1275 (0.88)	1.71 (1.61–1.81)	3.17 (2.94–3.41)	1.96 (1.74–2.21)	0.70 (0.62–0.79)
	1000–1499 g	8931 (0.64)	2108 (1.45)	2.27 (2.16–2.38)	3.74 (3.51–3.98)	2.78 (2.54–3.05)	1.16 (1.06–1.26)
	1500–1999 g	20,765 (1.49)	4906 (3.38)	2.27 (2.20–2.34)	3.13 (2.99–3.27)	3.13 (2.96–3.31)	1.41 (1.34–1.49)
	2000–2499 g	73,067 (5.23)	11,402 (7.85)	1.50 (1.47–1.53)	1.65 (1.60–1.70)	2.29 (2.22–2.37)	1.11 (1.08–1.14)
	>2499 g	1,286,667 (92.13)	125,632 (86.45)	0.94 (0.94–0.94)	0.90 (0.89–0.90)	0.87 (0.87–0.88)	0.99 (0.99–0.99)
Surgery	ref N = 1,389,446 ^b^						
	No	1,388,540 (99.93)	143,853 (99.86)	1.00 (1.00–1.00)	1.00 (1.00–1.00)	1.00 (1.00–1.00)	1.00 (1.00–1.00)
	Minor abdominal	75 (0.01)	20 (0.01)	2.57 (1.57–4.21)	3.31 (1.60–6.86)	4.83 (2.22–10.47)	1.28 (0.52–3.16)
	Major surgery	831 (0.06)	188 (0.13)	2.18 (1.86–2.56)	3.02 (2.41–3.80)	2.18 (1.55–3.05)	1.66 (1.31–2.12)
DRG-embedded complication level ^d^	ref N = 1,379,721 ^b^						
	None	647,136 (46.90)	17,447 (12.31)	0.26 (0.26–0.27)	0.59 (0.58–0.60)	0.44 (0.43–0.45)	0.00 (0.00–0.00)
	Moderate	434,550 (31.50)	107,377 (75.74)	2.40 (2.40–2.41)	1.47 (1.46–1.49)	2.19 (2.17–2.21)	3.05 (3.04–3.06)
	Severe	25,182 (1.83)	5969 (4.21)	2.31 (2.24–2.37)	2.83 (2.71–2.95)	2.00 (1.88–2.13)	2.10 (2.02–2.19)
	Other	272,853 (19.78)	10,981 (7.75)	0.39 (0.38–0.40)	1.06 (1.04–1.08)	0.35 (0.33–0.36)	0.00 (0.00–0.00)
Ventilation	ref N = 1,400,868 ^b^						
	No	1,393,049 (99.44)	142,737 (98.05)	0.99 (0.99–0.99)	0.98 (0.98–0.99)	0.99 (0.99–0.99)	0.99 (0.98–0.99)
	≤5 days	6373 (0.45)	2076 (1.43)	3.13 (2.98–3.29)	3.40 (3.14–3.67)	2.40 (2.13–2.69)	3.25 (3.04–3.46)
	>5 days	1446 (0.10)	758 (0.52)	5.04 (4.62–5.51)	5.82 (5.12–6.62)	3.81 (3.13–4.63)	5.02 (4.49–5.62)

^a^ where P00, P04 or P70.0-P70.1 is coded. ^b^ N values in the column headers represent the total number of eligible episodes in each cohort. For specific variables (e.g., birth weight tiers, surgical intervention, or comorbidity levels), denominators vary slightly because certain DRG categories do not embed explicit information for that attribute and were treated as missing. All PRs and 95% Confidence Intervals were computed based on available valid cases within each domain. ^c^ Calculated by the authors based on the available outcome variables (birth weight, problem severity, surgery, ventilation) ^d^ Native, statutory comorbidity modifier embedded directly into the official h-DRG grouper nomenclature, where modifiers are triggered automatically by the national health insurance algorithm based on extensive, predefined lists of secondary ICD-10 diagnoses attached to the hospitalization. † Haldane–Anscombe 0.5 correction applied (zero cell).

## Data Availability

The data presented in this study are available on request from the corresponding author. (Data are not publicly available due to privacy restrictions.).

## Supplementary Materials

The following supporting information can be downloaded at https://www.mdpi.com/article/10.3390/medsci14050591/s1, Table S1: Description of severity levels categorized by h-DRG labels; Table S2: Changes of main outcomes regarding composite clinical severity categories for each individual ICD-10 code from 2010 to 2025; Table S3: Detailed results of the joinpoint regression analyses; Table S4: Parallel and sensitivity analyses.

## Abbreviations

The following abbreviations are used in this manuscript:

AAPC	Annual average percentage change
APC	Annual percentage change
ENT	Ear, nose, and throat
GDM	Gestational diabetes mellitus
h-DRG	Hungarian Diagnosis Related Group
ICD	International Classification of Diseases
KSH	Hungarian Central Statistical Office
LBW	Low birth weight
LOS	Length of stay
NAS	Neonatal abstinence syndrome
NICU	Neonatal Intensive Care Unit
PAPP-A	Pregnancy-Associated Plasma Protein-A
PR	Prevalence ratio
