# Supplementary material for: Neonatal Outcomes Associated with Maternal-Factor-Related Diagnoses: Real-World Evidence from a National Inpatient Database (2010–2025)
Source: Med Sci (Basel). 2026 Sep 19;14(5):591. doi: 10.3390/medsci14050591 (PMC13609305; doi:10.3390/medsci14050591)
Supplement: Supplementary file 1 [file medsci-14-00591-s001.zip › Table S1_20260908.pdf]

Table S1. Description of severity levels categorized by h-DRG labels.

DRG code	DRG label	Severity	Justification
7300	Newborn, transferred before 5 days of age, born in the same hospital	2	Extremely low birth weight. Requires neonatal intensive care by definition and carries the highest neonatal mortality and morbidity of all birth-weight strata, irrespective of any additional coded condition.
7310	Newborn, transferred before 5 days of age, born in another hospital	2	Very low birth weight combined with major surgery: the highest possible combination of physiological immaturity and invasive intervention, always managed in a tertiary neonatal intensive care setting.
7130	Newborn, birth weight 1000–1499 g, without major surgery	3	Very low birth weight (<1500 g) is per se a critical condition requiring intensive or high-dependency neonatal care, prolonged hospitalisation and respiratory, thermal and nutritional support; it was therefore classified as severe independently of additional coded problems.
7120	Newborn, birth weight 1000–1499 g, with major surgery	3	Major surgical intervention in a low-birth-weight neonate implies perioperative intensive care and a substantial risk of complications; surgery was treated as a severity-defining criterion throughout the framework.
7170	Newborn, birth weight 1500–1999 g, without major surgery, with other problems	2	The official DRG label explicitly designates a severe problem; combined with low birth weight this denotes high care intensity.
7160	Newborn, birth weight 1500–1999 g, without major surgery, with moderate problems	2	The label explicitly designates a moderate-level problem. Low birth weight with a moderate coded problem requires increased monitoring and prolonged stay but does not, by definition, denote a critical condition.
715Z	Newborn, birth weight 1500–1999 g, without major surgery, with severe problems	3	Low birth weight accompanied by an unspecified (“other”) coded problem. Because the label does not identify a critical condition, the episode was classified as moderate rather than severe, in order to avoid overstating severity.
7140	Newborn, birth weight 1500–1999 g, with major surgery	3	Major surgery is a severity-defining criterion; combined with low birth weight it implies perioperative intensive care.
719Z	Newborn, birth weight 2000–2499 g, without major surgery, with severe problems	3	The official label explicitly designates a severe problem.
7220	Newborn, birth weight 2000–2499 g, without major surgery, with other problems	2	The label explicitly designates a moderate-level problem in a low-birth-weight neonate: increased care intensity without a documented critical condition.
7200	Newborn, birth weight 2000–2499 g, without major surgery, with moderate problems	2	Low birth weight is present, but the label explicitly states a normal newborn diagnosis, i.e. no coded morbidity. The episode was therefore classified as mild: birth weight below 2500 g justifies a non-zero category, while the absence of any coded problem precludes a higher one.
7210	Newborn, birth weight 2000–2499 g, without major surgery, with normal newborn diagnosis	1	Low birth weight with an unspecified coded problem; increased care intensity but no documented critical condition.
7180	Newborn, birth weight 2000–2499 g, with major surgery	3	Major surgery in the neonatal period is a severity-defining criterion irrespective of birth weight, implying operative and postoperative intensive care.
736Z	Newborn, birth weight ≥2500 g, without major surgery, with severe problems, with mechanical ventilation >5 days and surfactant therapy	3	A surgical intervention of limited extent. It denotes increased care intensity and prolonged hospitalisation, but not the physiological instability associated with major surgery; classified as moderate to preserve the distinction between minor and major procedures.
735Z	Newborn, birth weight ≥2500 g, without major surgery, with severe problems, with mechanical ventilation >5 days or surfactant therapy	3	The official label explicitly designates a moderate-level problem, requiring increased observation or treatment in a term neonate of normal birth weight.
734Z	Newborn, birth weight ≥2500 g, without major surgery, with severe problems, with mechanical ventilation ≤5 days	3	Term neonate of normal birth weight with an explicitly normal newborn diagnosis: routine postnatal care with no coded morbidity. This DRG defines the reference state of the classification.

7280	Newborn, birth weight ≥2500 g, without major surgery, with other problems	2	An unspecified ("other") coded problem in a neonate of normal birth weight. The presence of a coded problem excludes category 0–1, while the absence of a specified critical condition excludes category 3.
7260	Newborn, birth weight ≥2500 g, without major surgery, with moderate problems	2	Transfer within the first five days of life indicates that the neonate could not remain on a routine postnatal ward and required escalation of care, additional diagnostic work-up or access to a higher-level neonatal service. However, the DRG itself does not specify any defined life-threatening condition; it was therefore classified as moderate in order not to conflate organisational transfer with documented severe morbidity.
7270	Newborn, birth weight ≥2500 g, without major surgery, with normal newborn diagnosis	0	Same rationale as h-DRG 7300. In addition, this DRG identifies an episode at a receiving institution, i.e. increased care intensity and inter-institutional escalation, without a specified critical diagnosis in the label.
7230	Newborn, birth weight ≥2500 g, with major surgery	3	The label explicitly designates a severe problem, and mechanical ventilation of any duration indicates intensive care with respiratory failure; both criteria independently define category 3.
7240	Newborn, birth weight ≥2500 g, with minor abdominal surgery	2	Prolonged mechanical ventilation or surfactant administration denotes established respiratory failure managed in a neonatal intensive care unit.
7110	Newborn, birth weight <999 g	3	The most resource-intensive non-surgical neonatal DRG: prolonged ventilation combined with surfactant therapy, i.e. severe respiratory failure requiring maximal intensive care.
