# Supplementary material for: Neonatal Outcomes Associated with Maternal-Factor-Related Diagnoses: Real-World Evidence from a National Inpatient Database (2010–2025)
Source: Med Sci (Basel). 2026 Sep 19;14(5):591. doi: 10.3390/medsci14050591 (PMC13609305; doi:10.3390/medsci14050591)
Supplement: Supplementary file 1 [file medsci-14-00591-s001.zip › Table S2_20260908.pdf]

Table S2. Changes of main outcomes regarding composite clinical severity categories for each individual ICD-10 code from 2010 to 2025.

Case number (n)	Disease groups	*None*			*Mild*			*Moderate*			*Severe*			*Total*		
		2010	2025	*Change (%)*	2010	2025	*Change (%)*	2010	2025	*Change (%)*	2010	2025	*Change (%)*	2010	2025	*Change (%)*
	Fetus and newborn affected by maternal hypertensive disorders (P00.0)	51	120	*135.29%*	5	3	*-40.00%*	229	739	*222.71%*	44	107	*143.18%*	329	969	*194.53%*
	Fetus and newborn affected by maternal renal and urinary tract diseases (P00.1)	2	1	*-50.00%*	-	-	-	14	2	*-85.71%*	-	-	-	16	3	*-81.25%*
	Fetus and newborn affected by maternal infectious and parasitic diseases (P00.2)	45	57	*26.67%*	2	4	*100.00%*	132	1256	*851.52%*	12	69	*475.00%*	191	1386	*625.65%*
	Fetus and newborn affected by other maternal circulatory and respiratory diseases (P00.3)	-	-	-	-	-	-	16	1	*-93.75%*	1	-	-	17	1	*-94.12%*
	Fetus and newborn affected by maternal nutritional disorders (P00.4)	3	-	-	-	-	-	2	1	*-50.00%*	-	-	-	5	1	*-80.00%*
	Fetus and newborn affected by maternal injury (P00.5)	-	-	-	-	-	-	-	-	-	-	1	-	-	1	-
	Fetus and newborn affected by surgical procedure on mother (P00.6)	-	-	-	-	-	-	-	-	-	-	-	-	-	-	-
	Fetus and newborn affected by other medical procedures on mother, not elsewhere classified (P00.7)	-	1	-	-	-	-	1	4	*300.00%*	-	-	-	1	5	*400.00%*
	Fetus and newborn affected by other maternal conditions (P00.8)	80	444	*455.00%*	11	11	*0.00%*	272	2194	*706.62%*	22	279	*1168.18%*	385	2928	*660.52%*
	Fetus and newborn affected by unspecified maternal condition (P00.9)	4	6	*50.00%*	2	-	-	19	14	*-26.32%*	1	3	*200.00%*	26	23	*-11.54%*
	**Fetus and newborn affected by maternal conditions that may be unrelated to present pregnancy (P00)**	185	629	*240.00%*	20	18	*-10.00%*	685	4211	*514.74%*	80	459	*473.75%*	970	5317	*448.14%*
	Fetus and newborn affected by maternal anaesthesia and analgesia in pregnancy, labour and delivery (P04.0)	1	2	*100.00%*	-	1	-	12	4	*-66.67%*	1	1	*0.00%*	14	8	*-42.86%*
	Fetus and newborn affected by other maternal medication (P04.1)	2		*-100.00%*	-	-	-	289	2552	*783.04%*	4	182	*4450.00%*	295	2734	*826.78%*
	Fetus and newborn affected by maternal use of tobacco (P04.2)	64	357	*457.81%*	10	12	*20.00%*	286	528	*84.62%*	26	38	*46.15%*	386	935	*142.23%*
	Fetus and newborn affected by maternal use of alcohol (P04.3)	-	-	-	1	-	-	1	-	-	1	1	*0.00%*	3	1	*-66.67%*
	Fetus and newborn affected by maternal use of drugs of addiction (P04.4)	-	-	-	-	-	-	5	11	*120.00%*	1	1	*0.00%*	6	12	*100.00%*
	Fetus and newborn affected by maternal use of nutritional chemical substances (P04.5)	-	-	-	-	-	-	2	-	-	1	-	-	3	-	-
	Fetus and newborn affected by maternal exposure to environmental chemical substances (P04.6)	-	-	-	-	-	-	-	-	-	-	-	-	-	-	-
	Fetus and newborn affected by other maternal noxious influences (P04.8)	3	1	*-66.67%*	-	-	-	2	46	*2200.00%*	-	9	-	5	56	*1020.00%*
	Fetus and newborn affected by maternal noxious influence, unspecified (P04.9)	-	1	-	-	-	-	-	228	-	-	45	-	-	274	-
	**Fetus and newborn affected by noxious influences transmitted via placenta or breast milk (P04)**	**70**	**361**	*415.71%*	**11**	**13**	*18.18%*	**597**	**3369**	*464.32%*	**34**	**277**	*714.71%*	**712**	**4020**	*464.61%*
	Syndrome of infant of mother with gestational diabetes (P70.0)	1	-	-	-	-	-	2158	4612	*113.72%*	95	267	*181.05%*	2254	4879	*116.46%*
	Syndrome of infant of a diabetic mother (P70.1)	-	-	-	-	-	-	266	334	*25.56%*	23	30	*30.43%*	289	364	*25.95%*
	**Syndrome of infant of mother with gestational diabetes or diabetes (P70)**	**1**	**-**	-	-	-	-	**2424**	**4946**	*104.04%*	**118**	**297**	*151.69%*	**2543**	**5243**	*106.17%*
	**Total**	**186**	**990**	*432.26%*	**31**	**31**	*0.00%*	**3706**	**12526**	*237.99%*	**232**	**1033**	*345.26%*	**4225**	**14580**	*245.09%*

Case	Disease groups	None			Mild			Moderate			Severe			Total		
		2010	2025	*Change (%)*	2010	2025	*Change (%)*	2010	2025	*Change (%)*	2010	2025	*Change (%)*	2010	2025	*Change (%)*
	Fetus and newborn affected by maternal hypertensive disorders (P00.0)	0.565	1.666	*195.14%*	0.055	0.042	*-24.74%*	2.535	10.261	*304.79%*	0.487	1.486	*205.04%*	3.642	13.455	*269.44%*

	Fetus and newborn affected by maternal renal and urinary tract diseases (P00.1)	0.022	0.014	*-37.28%*	-	-	-	0.155	0.028	*-82.08%*	-	-	-	0.177	0.042	*-76.48%*
	Fetus and newborn affected by maternal infectious and parasitic diseases (P00.2)	0.498	0.791	*58.89%*	0.022	0.056	*150.87%*	1.461	17.440	*1093.54%*	0.133	0.958	*621.26%*	2.114	19.245	*810.23%*
	Fetus and newborn affected by other maternal circulatory and respiratory diseases (P00.3)	-	-	-	-	-	-	0.177	0.014	*-92.16%*	0.011	-	-	0.188	0.014	*-92.62%*
	Fetus and newborn affected by maternal nutritional disorders (P00.4)	0.033	-	-	-	-	-	0.022	0.014	*-37.28%*	-	-	-	0.055	0.014	*-74.91%*
	Fetus and newborn affected by maternal injury (P00.5)	-	-	-	-	-	-	-	-	-	-	0.014	-	-	0.014	-
	Fetus and newborn affected by surgical procedure on mother (P00.6)	-	-	-	-	-	-	-	-	-	-	-	-	-	-	-
	Fetus and newborn affected by other medical procedures on mother, not elsewhere classified (P00.7)	-	0.014	-	-	-	-	0.011	0.056	*401.74%*	-	-	-	0.011	0.069	*527.18%*
	Fetus and newborn affected by other maternal conditions (P00.8)	0.886	6.165	*596.17%*	0.122	0.153	*25.44%*	3.011	30.465	*911.79%*	0.244	3.874	*1490.75%*	4.262	40.657	*853.96%*
	Fetus and newborn affected by unspecified maternal condition (P00.9)	0.044	0.083	*88.15%*	0.022	-	-	0.210	0.194	*-7.57%*	0.011	0.042	*276.31%*	0.288	0.319	*10.96%*
	**Fetus and newborn affected by maternal conditions that may be unrelated to present pregnancy (P00)**	2.048	8.734	*326.48%*	0.221	0.250	*12.89%*	7.583	58.472	*671.11%*	0.886	6.373	*619.69%*	10.738	73.830	*587.57%*
	Fetus and newborn affected by maternal anaesthesia and analgesia in pregnancy, labour and delivery (P04.0)	0.011	0.028	*150.87%*	-	0.014	-	0.133	0.056	*-58.19%*	0.011	0.014	*25.44%*	0.155	0.111	*-28.32%*
	Fetus and newborn affected by other maternal medication (P04.1)	0.022	0.000	*-100.00%*	-	-	-	3.199	35.436	*1007.65%*	0.044	2.527	*5607.32%*	3.266	37.963	*1062.51%*
	Fetus and newborn affected by maternal use of tobacco (P04.2)	0.708	4.957	*599.70%*	0.111	0.167	*50.52%*	3.166	7.332	*131.57%*	0.288	0.528	*83.33%*	4.273	12.983	*203.84%*
	Fetus and newborn affected by maternal use of alcohol (P04.3)	-	-	-	0.011	-	-	0.011	-	-	0.011	0.014	*25.44%*	0.033	0.014	*-58.19%*
	Fetus and newborn affected by maternal use of drugs of addiction (P04.4)	-	-	-	-	-	-	0.055	0.153	*175.96%*	0.011	0.014	*25.44%*	0.066	0.167	*150.87%*
	Fetus and newborn affected by maternal use of nutritional chemical substances (P04.5)	-	-	-	-	-	-	0.022	-	-	0.011	-	-	0.033	-	-
	Fetus and newborn affected by maternal exposure to environmental chemical substances (P04.6)	-	-	-	-	-	-	-	-	-	-	-	-	-	-	-
	Fetus and newborn affected by other maternal noxious influences (P04.8)	0.033	0.014	*-58.19%*	-	-	-	0.022	0.639	*2785.02%*	-	0.125	-	0.055	0.778	*1304.88%*
	Fetus and newborn affected by maternal noxious influence, unspecified (P04.9)	-	0.014	-	-	-	-	-	3.166	-	-	0.625	-	-	3.805	-
	**Fetus and newborn affected by noxious influences transmitted via placenta or breast milk (P04)**	0.775	5.013	*546.89%*	0.122	0.181	*48.24%*	6.609	46.781	*607.86%*	0.376	3.846	*921.93%*	7.882	55.820	*608.22%*
	Syndrome of infant of mother with gestational diabetes (P70.0)	0.011	-	-	-	-	-	23.889	64.040	*168.08%*	1.052	3.707	*252.54%*	24.952	67.748	*171.52%*
	Syndrome of infant of a diabetic mother (P70.1)	-	-	-	-	-	-	2.945	4.638	*57.50%*	0.255	0.417	*63.61%*	3.199	5.054	*57.99%*
	**Syndrome of infant of mother with gestational diabetes or diabetes (P70)**	0.011	-	-	-	-	-	26.833	68.678	*155.94%*	1.306	4.124	*215.72%*	28.151	72.802	*158.62%*
	**Total**	2.059	13.747	*567.64%*	0.343	0.430	*25.44%*	41.025	173.931	*323.96%*	2.568	14.344	*458.51%*	46.770	202.452	*332.86%*

	**Disease groups**	**None**			**Mild**			**Moderate**			**Severe**			**Total**		
		**2010**	**2025**	***Change (%)***	**2010**	**2025**	***Change (%)***	**2010**	**2025**	***Change (%)***	**2010**	**2025**	***Change (%)***	**2010**	**2025**	***Change (%)***
**DRG weight (amount)**	Fetus and newborn affected by maternal hypertensive disorders (P00.0)	16.09	48.93	*204.10%*	1.98	2.05	*3.54%*	116.3	530.66	*356.29%*	357.51	886.76	*148.04%*	491.88	1468.4	*198.53%*
	Fetus and newborn affected by maternal renal and urinary tract diseases (P00.1)	0.35	0.47	*34.29%*	-	-	-	9.6	1.88	*-80.42%*	-	-	-	9.95	2.35	*-76.38%*
	Fetus and newborn affected by maternal infectious and parasitic diseases (P00.2)	12.8	24.17	*88.83%*	0.16	2.5	*1462.50%*	58.51	860.94	*1371.44%*	55.37	404.45	*630.45%*	126.84	1292.06	*918.65%*
	Fetus and newborn affected by other maternal circulatory and respiratory diseases (P00.3)	-	-	-	-	-	-	9.02	0.56	*-93.79%*	-	-	-	9.02	0.56	*-93.79%*
	Fetus and newborn affected by maternal nutritional disorders (P00.4)	0.63	-	-	-	-	-	0.46	1.51	*228.26%*	-	-	-	1.09	1.51	*38.53%*

Fetus and newborn affected by maternal injury (P00.5)	-	-	-	-	-	-	-	-	-	-	5.37	-	-	5.37	-
Fetus and newborn affected by surgical procedure on mother (P00.6)	-	-	-	-	-	-	-	-	-	-	-	-	-	-	-
Fetus and newborn affected by other medical procedures on mother, not elsewhere classified (P00.7)	-	0.37	-	-	-	-	0.52	1.1	*111.54%*	-	-	-	0.52	1.47	*182.69%*
Fetus and newborn affected by other maternal conditions (P00.8)	24.61	183.87	*647.14%*	4.67	6.86	*46.90%*	104.19	1339.98	*1186.09%*	79.13	1233.75	*1459.14%*	212.6	2764.46	*1200.31%*
Fetus and newborn affected by unspecified maternal condition (P00.9)	1.26	2.38	*88.89%*	0.95	-	-	7.56	9.55	*26.32%*	11.49	1.6	*-86.07%*	21.26	13.53	*-36.36%*
**Fetus and newborn affected by maternal conditions that may be unrelated to present pregnancy (P00)**	55.74	260.19	*366.79%*	7.76	11.41	*47.04%*	306.16	2746.18	*796.98%*	503.5	2531.93	*402.87%*	873.16	5549.71	*535.59%*
Fetus and newborn affected by maternal anaesthesia and analgesia in pregnancy, labour and delivery (P04.0)	0.32	0.84	*162.50%*	-	0.71	-	5.41	2.88	*-46.77%*	3.27	-	-	9	4.43	*-50.78%*
Fetus and newborn affected by other maternal medication (P04.1)	0.24	-	-	-	-	-	136.91	1862.27	*1260.21%*	17.81	893.29	*4915.67%*	154.96	2755.56	*1678.24%*
Fetus and newborn affected by maternal use of tobacco (P04.2)	20.11	145.49	*623.47%*	3.48	8.31	*138.79%*	151.04	367.28	*143.17%*	178.4	373.71	*109.48%*	353.03	894.79	*153.46%*
Fetus and newborn affected by maternal use of alcohol (P04.3)	-	-	-	-	-	-	1.46	-	-	-	15.05	-	1.46	15.05	*930.82%*
Fetus and newborn affected by maternal use of drugs of addiction (P04.4)	-	-	-	-	-	-	1.57	9.04	*475.80%*	8.24	3.79	*-54.00%*	9.81	12.83	*30.78%*
Fetus and newborn affected by maternal use of nutritional chemical substances (P04.5)	-	-	-	-	-	-	0.32	-	-	-	-	-	0.32	-	-
Fetus and newborn affected by maternal exposure to environmental chemical substances (P04.6)	-	-	-	-	-	-	-	-	-	-	-	-	-	-	-
Fetus and newborn affected by other maternal noxious influences (P04.8)	0.95	0.47	*-50.53%*	-	-	-	0.89	33.56	*3670.79%*	-	27.26	-	1.84	61.29	*3230.98%*
Fetus and newborn affected by maternal noxious influence, unspecified (P04.9)	-	0.47	-	-	-	-	-	133.1	-	-	195.04	-	-	328.61	-
**Fetus and newborn affected by noxious influences transmitted via placenta or breast milk (P04)**	**21.62**	**147.27**	*581.17%*	**3.48**	**9.02**	*159.20%*	**297.6**	**2408.13**	*709.18%*	**207.72**	**1508.14**	*626.04%*	**530.42**	**4072.56**	*667.80%*
Syndrome of infant of mother with gestational diabetes (P70.0)	0.32	-	-	-	-	-	1031.56	2929.04	*183.94%*	420.37	972.49	*131.34%*	1452.25	3901.53	*168.65%*
**Syndrome of infant of mother with gestational diabetes or diabetes (P70)**	-	-	-	-	-	-	124.79	210.65	*68.80%*	64.25	57.13	*-11.08%*	189.04	267.78	*41.65%*
**Total**	**0.32**	**-**	-	**-**	**-**	-	**1156.35**	**3139.69**	*171.52%*	**484.62**	**1029.62**	*112.46%*	**1641.29**	**4169.31**	*154.03%*

	Disease groups	None			Mild			Moderate			Severe			Total		
		**2010**	**2025**	***Change (%)***	**2010**	**2025**	***Change (%)***	**2010**	**2025**	***Change (%)***	**2010**	**2025**	***Change (%)***	**2010**	**2025**	***Change (%)***
**Mean LOS (days)**	Fetus and newborn affected by maternal hypertensive disorders (P00.0)	4.37	2.38	*-45.68%*	6.80	3.00	*-55.88%*	4.78	3.73	*-21.85%*	8.95	6.28	*-29.86%*	5.30	3.84	*-27.52%*
	Fetus and newborn affected by maternal renal and urinary tract diseases (P00.1)	2.00	4.00	*100.00%*	-	-	-	6.07	6.00	*-1.18%*	-	-	-	5.56	5.33	*-4.12%*
	Fetus and newborn affected by maternal infectious and parasitic diseases (P00.2)	4.18	3.07	*-26.51%*	0.50	2.75	*450.00%*	5.00	3.75	*-25.00%*	4.42	7.01	*58.82%*	4.72	3.88	*-17.80%*
	Fetus and newborn affected by other maternal circulatory and respiratory diseases (P00.3)	-	-	-	-	-	-	5.00	3.00	*-40.00%*	-	-	-	4.71	3.00	*-36.25%*
	Fetus and newborn affected by maternal nutritional disorders (P00.4)	2.33	-	-	-	-	-	2.00	12.00	*500.00%*	-	-	-	2.20	12.00	*445.45%*
	Fetus and newborn affected by maternal injury (P00.5)	-	-	-	-	-	-	-	-	-	-	1.00	-	-	1.00	-
	Fetus and newborn affected by surgical procedure on mother (P00.6)	-	-	-	-	-	-	-	-	-	-	-	-	-	-	-
	Fetus and newborn affected by other medical procedures on mother, not elsewhere classified (P00.7)	-	2.00	-	-	-	-	7.00	1.25	*-82.14%*	-	-	-	7.00	1.40	*-80.00%*
	Fetus and newborn affected by other maternal conditions (P00.8)	4.13	2.84	*-31.20%*	6.09	2.73	*-55.22%*	4.21	4.33	*2.80%*	3.91	4.32	*10.58%*	4.23	4.10	*-3.12%*
	Fetus and newborn affected by unspecified maternal condition (P00.9)	4.50	2.33	*-48.15%*	3.00	-	-	4.00	4.00	*0.00%*	22.00	-	*#ÉRTÉK!*	4.69	3.04	*-35.14%*

**Fetus and newborn affected by maternal conditions that may be unrelated to present pregnancy (P00)**	4.16	2.77	*-33.54%*	5.40	2.78	*-48.56%*	4.60	4.05	*-11.97%*	6.94	5.15	*-25.79%*	4.73	3.99	*-15.59%*
Fetus and newborn affected by maternal anaesthesia and analgesia in pregnancy, labour and delivery (P04.0)	4.00	3.00	*-25.00%*	-	3.00	-	4.58	6.50	*41.82%*	10.00	-	*#ÉRTÉK!*	4.93	4.38	*-11.23%*
Fetus and newborn affected by other maternal medication (P04.1)	1.50	-	-	-	-	-	3.87	3.94	*1.97%*	4.75	6.23	*31.17%*	3.86	4.10	*6.02%*
Fetus and newborn affected by maternal use of tobacco (P04.2)	3.58	2.74	*-23.44%*	2.70	2.92	*8.02%*	4.28	4.96	*15.81%*	6.96	10.58	*51.96%*	4.30	4.31	*0.21%*
Fetus and newborn affected by maternal use of alcohol (P04.3)	-	-	-	-	-	-	1.00	-	-	-	12.00	-	0.33	12.00	*3500.00%*
Fetus and newborn affected by maternal use of drugs of addiction (P04.4)	-	-	-	-	-	-	3.00	8.18	*172.73%*	32.00	7.00	*-78.13%*	7.83	8.08	*3.19%*
Fetus and newborn affected by maternal use of nutritional chemical substances (P04.5)	-	-	-	-	-	-	2.50	-	-	-	-	-	1.67	-	-
Fetus and newborn affected by maternal exposure to environmental chemical substances (P04.6)	-	-	-	-	-	-	-	-	-	-	-	-	-	-	-
Fetus and newborn affected by other maternal noxious influences (P04.8)	4.00	4.00	*0.00%*	-	-	-	8.00	9.41	*17.66%*	-	5.11	-	5.60	8.63	*54.02%*
Fetus and newborn affected by maternal noxious influence, unspecified (P04.9)	-	3.00	-	-	-	-	-	10.30	-	-	6.49	-	-	9.65	-
**Fetus and newborn affected by noxious influences transmitted via placenta or breast milk (P04)**	3.54	2.75	*-22.52%*	2.45	2.92	-	4.08	4.62	*13.44%*	7.12	6.83	*-3.99%*	4.14	4.60	*11.05%*
Syndrome of infant of mother with gestational diabetes (P70.0)	3.00	-	-	-	-	-	3.87	3.01	*-22.10%*	6.83	4.37	*-35.97%*	3.99	3.09	*-22.66%*
Syndrome of infant of a diabetic mother (P70.1)	-	-	-	-	-	-	4.42	3.06	*-30.66%*	4.00	2.77	*-30.83%*	4.38	3.04	*-30.69%*
**Syndrome of infant of mother with gestational diabetes or diabetes (P70)**	3.00	-	-	-	-	-	3.93	3.01	*-23.21%*	6.28	4.21	*-32.92%*	4.03	3.08	*-23.60%*
**Total**	5.49	2.76	*-49.75%*	4.35	2.84	*-34.81%*	4.08	3.80	*-6.86%*	6.63	5.33	*-19.58%*	4.21	3.83	*-9.02%*
