# Supplementary material for: Neonatal Outcomes Associated with Maternal-Factor-Related Diagnoses: Real-World Evidence from a National Inpatient Database (2010–2025)
Source: Med Sci (Basel). 2026 Sep 19;14(5):591. doi: 10.3390/medsci14050591 (PMC13609305; doi:10.3390/medsci14050591)
Supplement: Supplementary file 1 [file medsci-14-00591-s001.zip › Table S3_20260908.pdf]

Table S3. Detailed results of the Joinpoint Regression Analyses

Severity / Disease group	Variable	AAPC (95%CI)	Trends					
			Trend 1		Trend 2		Trend 3	
			APC (95%CI)	Period	APC (95%CI)	Period	APC (95%CI)	Period
None	**Case number**	7.47*** (5.42 to 9.55)	13.32 (9.08 to 17.72)*	2010-2018	52.09 (31.71 to 75.62)*	2018-2020	-14.08 (-19.88 to -7.86)*	2020-2025
	**Cases per 1000 livebirths**	8.82*** (6.77 to 10.91)	13.12 (8.93 to 17.47)*	2010-2018	48.72 (28.98 to 71.48)*	2018-2020	-9.74 (-15.78 to -3.27)*	2020-2025
	**DRG weight**	9.19*** (7.34 to 11.07)	13.70 (9.91 to 17.62)*	2010-2018	56.31 (37.53 to 77.64)*	2018-2020	-11.34 (-16.69 to -5.65)*	2020-2025
	**LOS**	-2.28*** (-2.71 to -1.86)	-1.86 (-2.57 to -1.16)*	2010-2019	-6.26 (-9.23 to -3.18)*	2019-2021	-1.20 (-3.18 to 0.83)	2021-2025
Mild	**Case number**	1.07 (-1.71 to 3.93)	5.72 (0.73 to 10.95)*	2010-2018	19.21 (8.18 to 31.36)*	2018-2022	-28.05 (-38.68 to -15.59)*	2022-2025
	**Cases per 1000 livebirths**	2.54 (-0.29 to 5.44)	5.42 (0.44 to 10.65)*	2010-2018	19.45 (8.38 to 31.65)*	2018-2022	-22.33 (-33.81 to -8.84)*	2022-2025
	**DRG weight**	4.52*** (2.24 to 6.84)	6.77 (1.48 to 12.33)*	2010-2016	17.31 (11.80 to 23.08)*	2016-2022	-20.50 (-29.19 to -10.73)*	2022-2025
	**LOS**	-1.82** (-3.18 to -0.45)	-9.37 (-19.18 to 1.63)	2010-2012	3.09 (-0.60 to 6.92)	2012-2017	-2.85 (-4.99 to -0.66)*	2017-2025
Moderate	**Case number**	8.44*** (7.55 to 9.34)	8.02 (6.99 to 9.05)*	2010-2021	2.07 (-3.90 to 8.42)	2021-2023	17.69 (8.84 to 27.27)*	2023-2025
	**Cases per 1000 livebirths**	9.99*** (9.12 to 10.87)	7.61 (6.78 to 8.44)*	2010-2023	26.81 (19.02 to 35.12)*	2023-2025	-	-
	**DRG weight**	10.86*** (9.92 to 11.80)	9.45 (8.38 to 10.54)*	2010-2021	-0.21 (-6.23 to 6.19)	2021-2023	32.09 (21.85 to 43.19)*	2023-2025
	**LOS**	-0.43** (-0.72 to -0.14)	-1.35 (-1.62 to -1.07)*	2010-2023	5.75 (3.36 to 8.20)*	2023-2025	-	
Severe	**Case number**	10.95*** (10.41 to 11.50)	8.24 (6.33 to 10.18)*	2010-2015	28.19 (23.59 to 32.97)*	2015-2017	8.69 (7.64 to 9.75)*	2017-2025
	**Cases per 1000 livebirths**	11.94*** (11.61 to 12.28)	7.73 (6.56 to 8.90)*	2010-2015	25.19 (22.42 to 28.03)*	2015-2017	11.50 (10.84 to 12.16)*	2017-2025

	**DRG weight**	9.10***<br>(7.85 to 10.37)	-12.89<br>(-20.53 to -4.52)*	2010-2012	23.08<br>(20.11 to 26.13)*	2012-2018	4.92<br>(2.72 to 7.16)*	2018-2025
	**LOS**	-2.02***<br>(-2.93 to -1.11)	-11.17<br>(-18.30 to -3.42)*	2010-2012	8.57<br>(3.94 to 13.41)*	2012-2015	-3.11<br>(-4.26 to -1.96)*	2015-2025

**Fetus and newborn affected by maternal conditions that may be unrelated to present pregnancy (P00)**	**Case number**	11.74***<br>(10.74 to 12.75)	9.17<br>(7.24 to 11.13)*	2010-2018	38.61<br>(29.62 to 48.23)*	2018-2020	6.40<br>(2.99 to 9.93)*	2020-2025
	**Cases per 1000 livebirths**	13.14***<br>(12.01 to 14.29)	8.98<br>(6.82 to 11.18)*	2010-2018	35.54<br>(25.70 to 46.15)*	2018-2020	11.77<br>(7.75 to 15.94)*	2020-2025
	**DRG weight**	12.05***<br>(10.64 to 13.49)	-8.81<br>(-17.01 to 0.20)	2010-2012	19.85<br>(17.60 to 22.15)*	2012-2020	9.26<br>(5.74 to 12.90)*	2020-2025
	**LOS**	-1.15***<br>(-1.76 to -0.54)	-0.25<br>(-1.47 to 0.98)	2010-2018	-8.73<br>(-12.86 to -4.40)*	2018-2020	0.58<br>(-1.66 to 2.87)	2020-2025
**Fetus and newborn affected by noxious influences transmitted via placenta or breast milk (P04)**	**Case number**	13.19***<br>(11.18 to 15.24)	16.34<br>(11.09 to 21.83)*	2010-2015	0.62<br>(-2.03 to 3.33)	2015-2023	69.30<br>(48.28 to 93.30)*	2023-2025
	**Cases per 1000 livebirths**	14.85***<br>(12.74 to 16.99)	15.37<br>(9.99 to 21.00)*	2010-2015	1.22<br>(-1.53 to 4.05)	2015-2023	88.21<br>(64.11 to 115.84)*	2023-2025
	**DRG weight**	15.94***<br>(13.68 to 18.25)	19.43<br>(14.49 to 24.59)*	2010-2016	2.13<br>(-1.35 to 5.74)	2016-2023	65.30<br>(42.08 to 92.31)*	2023-2025
	**LOS**	0.46*<br>(0.03 to 0.89)	0.46<br>(0.03 to 0.89)*	2010-2025	-	-	-	-
**Transitory disorders of carbohydrate metabolism specific to fetus and newborn (P70)**	**Case number**	4.10***<br>(3.37 to 4.83)	6.28<br>(4.12 to 8.49)*	2010-2015	9.11<br>(7.43 to 10.81)*	2015-2021	-5.46 (-7.97 to -2.89)*	2021-2025
	**Cases per 1000 livebirths**	5.80***<br>(5.04 to 6.56)	7.55<br>(6.52 to 8.59)*	2010-2021	1.13<br>(-1.87 to 4.22)	2021-2025	-	-
	**DRG weight**	5.21***<br>(4.28 to 6.15)	2.96<br>(-0.49 to 6.52)	2010-2014	13.58<br>(11.38 to 15.83)*	2014-2020	-2.35<br>(-4.86 to 0.22)	2020-2025
	**LOS**	-1.45***<br>(-1.78 to -1.11)	-1.64<br>(-2.19 to -1.08)*	2010-2019	-4.40<br>(-6.78 to -1.96)*	2019-2021	0.50<br>(-1.08 to 2.10)	2021-2025
**Total**	**Case number**	8.52***<br>(7.67 to 9.37)	9.34<br>(8.35 to 10.34)*	2010-2021	1.17<br>(-4.46 to 7.14)	2021-2023	11.67<br>(3.66 to 20.30)*	2023-2025
	**Cases per 1000 livebirths**	9.98***<br>(9.12 to 10.85)	8.72<br>(7.90 to 9.55)*	2010-2023	18.52<br>(11.32 to 26.20)*	2023-2025	-	-

	**DRG weight**	9.41***<br>(8.42 to 10.41)	4.18<br>(-0.69 to 9.28)	2010-2013	14.64<br>(12.38 to 16.93)*	2013-2019	7.02<br>(4.79 to 9.29)*	2019-2025
	**LOS**	-0.59***<br>(-0.90 to -0.28)	-0.91<br>(-1.42 to -0.40)*	2010-2019	-5.16<br>(-7.34 to -2.94)*	2019-2021	2.54<br>(1.06 to 4.04)*	2021-2025
