# Supplementary material for: Neonatal Outcomes Associated with Maternal-Factor-Related Diagnoses: Real-World Evidence from a National Inpatient Database (2010–2025)
Source: Med Sci (Basel). 2026 Sep 19;14(5):591. doi: 10.3390/medsci14050591 (PMC13609305; doi:10.3390/medsci14050591)
Supplement: Supplementary file 1 [file medsci-14-00591-s001.zip › Table S4_20260908.pdf]

Table S4. Parallel and Sensitivity analyses

Parallel analysis: P70.2–P70.9 (non-maternal carbohydrate disorders) vs clean reference

Outcome variable	Level	P70.2–.9 n (%)	Clean reference n (%)	PR (95% CI)	All-Maternal PR (for comparison)
**Severity category**	**ref N = 1,290,799**				
	None	95 (0.09)	629,238 (48.75)	0.00 (0.00–0.00)	0.23 (0.23–0.24)
	Mild	38 (0.03)	17,765 (1.38)	0.03 (0.02–0.03)	0.41 (0.38–0.43)
	Moderate	97,799 (88.85)	613,926 (47.56)	1.87 (1.86–1.87)	1.71 (1.71–1.72)
	Severe	12,137 (11.03)	29,870 (2.31)	4.77 (4.67–4.86)	2.82 (2.76–2.89)
**Birth weight**	**ref N = 1,286,965**				
	<999 g	2,583 (2.36)	4,592 (0.36)	6.60 (6.29–6.93)	2.46 (2.31–2.62)
	1000-1499 g	2,973 (2.71)	5,958 (0.46)	5.86 (5.61–6.12)	3.13 (2.98–3.29)
	1500-1999 g	8,007 (7.30)	12,758 (0.99)	7.37 (7.17–7.57)	3.41 (3.30–3.52)
	2000-2499 g	20,320 (18.53)	52,747 (4.10)	4.52 (4.45–4.59)	1.91 (1.88–1.95)
	>2499 g	75,757 (69.10)	1,210,910 (94.09)	0.73 (0.73–0.74)	0.92 (0.92–0.92)
**Surgery**	**ref N = 1,282,562**				
	No	106,862 (99.98)	1,281,678 (99.93)	1.00 (1.00–1.00)	1.00 (1.00–1.00)
	Minor abdominal	8 (0.01)	67 (0.01)	1.43 (0.69–2.98)	2.66 (1.61–4.38)
	Major surgery	14 (0.01)	817 (0.06)	0.21 (0.12–0.35)	2.05 (1.75–2.40)
**DRG-embedded complication level**	**ref N = 1,275,783**				
	None	133 (0.13)	647,003 (50.71)	0.00 (0.00–0.00)	0.24 (0.24–0.25)
	Moderate	88,848 (85.48)	345,702 (27.10)	3.15 (3.14–3.17)	2.80 (2.78–2.81)
	Severe	6,439 (6.20)	18,743 (1.47)	4.22 (4.10–4.33)	2.87 (2.78–2.95)
	Other	8,518 (8.20)	264,335 (20.72)	0.40 (0.39–0.40)	0.37 (0.37–0.38)
**Ventilation**	**ref N = 1,290,799**				
	No	107,689 (97.84)	1,285,360 (99.58)	0.98 (0.98–0.98)	0.98 (0.98–0.99)
	<=5 days	1,767 (1.61)	4,606 (0.36)	4.50 (4.26–4.75)	4.00 (3.80–4.21)
	>5 days	613 (0.56)	833 (0.06)	8.63 (7.78–9.58)	8.07 (7.31–8.90)

P70.2–P70.9 were not treated as maternal-factor exposure. Reported separately, these episodes had a more severe recorded profile than P70.0–P70.1, which supports excluding them from the primary definition rather than pooling the whole P70 block.

Sensitivity analysis: effect of the reference-group definition

Outcome variable	Level	All-Maternal PR main (ref 1,400,868)	All-Maternal PR clean ref (P70.2–.9 excl.)
**Severity category**	**clean ref N = 1,290,799**		
	None	0.25 (0.25–0.26)	0.23 (0.23–0.24)
	Mild	0.44 (0.41–0.47)	0.41 (0.38–0.43)
	Moderate	1.60 (1.60–1.61)	1.71 (1.71–1.72)
	Severe	2.18 (2.13–2.23)	2.82 (2.76–2.89)
**Birth weight**	**clean ref N = 1,286,965**		
	<999 g	1.71 (1.61–1.81)	2.46 (2.31–2.62)
	1000-1499 g	2.27 (2.16–2.38)	3.13 (2.98–3.29)
	1500-1999 g	2.27 (2.20–2.34)	3.41 (3.30–3.52)
	2000-2499 g	1.50 (1.47–1.53)	1.91 (1.88–1.95)
	>2499 g	0.94 (0.94–0.94)	0.92 (0.92–0.92)
**Surgery**	**clean ref N = 1,282,562**		
	No	1.00 (1.00–1.00)	1.00 (1.00–1.00)
	Minor abdominal	2.57 (1.57–4.21)	2.66 (1.61–4.38)
	Major surgery	2.18 (1.86–2.56)	2.05 (1.75–2.40)
**DRG-embedded complication level**	**clean ref N = 1,275,783**		
	None	0.26 (0.26–0.27)	0.24 (0.24–0.25)
	Moderate	2.40 (2.40–2.41)	2.80 (2.78–2.81)
	Severe	2.31 (2.24–2.37)	2.87 (2.78–2.95)
	Other	0.39 (0.38–0.40)	0.37 (0.37–0.38)
**Ventilation**	**clean ref N = 1,290,799**		
	No	0.99 (0.99–0.99)	0.98 (0.98–0.99)
	<=5 days	3.13 (2.98–3.29)	4.00 (3.80–4.21)
	>5 days	5.04 (4.62–5.51)	8.07 (7.31–8.90)

Main analysis keeps P70.2–P70.9 in the reference (conservative, standard convention). The clean reference removes them, testing dilution from suspected under-coded maternal diabetes.
